# Human Activity Recognition Data Analysis: History, Evolutions, and New Trends

**DOI:** 10.3390/s22093401

**Published:** 2022-04-29

**Authors:** Paola Patricia Ariza-Colpas, Enrico Vicario, Ana Isabel Oviedo-Carrascal, Shariq Butt Aziz, Marlon Alberto Piñeres-Melo, Alejandra Quintero-Linero, Fulvio Patara

**Affiliations:** 1 Department of Computer Science and Electronics, Universidad de la Costa CUC, Barranquilla 080002, Colombia; 2 Faculty of Engineering in Information and Communication Technologies, Universidad Pontificia Bolivariana, Medellín 050031, Colombia; ana.oviedo@upb.edu.co; 3 Department of Information Engineering, University of Florence, 50139 Firenze, Italy; enrico.vicario@unifi.it (E.V.); fulvio.patara@unifi.it (F.P.); 4 Department of Computer Science and IT, University of Lahore, Lahore 44000, Pakistan; shariq2315@gmail.com; 5 Department of Systems Engineering, Universidad del Norte, Barranquilla 081001, Colombia; pineresm@uninorte.edu.co; 6 Microbiology Program, Universidad Popular del Cesar, Valledupar 200002, Colombia; alejandraquinterol@unicesar.edu.co

**Keywords:** ambient assisted living—AAL, human activity recognition—HAR, activities of daily living—ADL, activity recognition systems—ARS, clustering, unsupervised activity recognition, supervised learning, unsupervised learning, ensemble learning, deep learning, reinforcement learning

## Abstract

The Assisted Living Environments Research Area–AAL (Ambient Assisted Living), focuses on generating innovative technology, products, and services to assist, medical care and rehabilitation to older adults, to increase the time in which these people can live. independently, whether they suffer from neurodegenerative diseases or some disability. This important area is responsible for the development of activity recognition systems—ARS (Activity Recognition Systems), which is a valuable tool when it comes to identifying the type of activity carried out by older adults, to provide them with assistance. that allows you to carry out your daily activities with complete normality. This article aims to show the review of the literature and the evolution of the different techniques for processing this type of data from supervised, unsupervised, ensembled learning, deep learning, reinforcement learning, transfer learning, and metaheuristics approach applied to this sector of science. health, showing the metrics of recent experiments for researchers in this area of knowledge. As a result of this article, it can be identified that models based on reinforcement or transfer learning constitute a good line of work for the processing and analysis of human recognition activities.

## 1. Introduction

Currently, the number of older adults who require a caregiver due to various conditions related to neurodegenerative diseases has greatly increased. This situation constitutes a great problem both for society and for integrated health systems worldwide because there is not enough infrastructure to be able to massively attend to the increasing number of people with this type of condition. Due to the above, a line of research arises that relates the sensory and the processing of HAR (Human Activity Recognition) data, which allows supporting the management of these individuals.

In general, this type of experimentation uses a model as a representation of reality developed to study it. In most analyzes it is not necessary to consider all the details of reality, the model is not only a substitute for reality but also a simplification of it. According to the same author, the models are classified as iconic, analogous, symbolic, deterministic, stochastic, static, continuous, and discrete depending on the tools used [1]. On the other hand, Cramér [2] from the foundation of probability theory, specifies from the object of probability theory that a mathematical model makes a description of a certain class of observed phenomena.

Artificial intelligence (AI) is defined as a field of science and engineering concerned with the computational understanding of what is commonly called intelligent behavior with the creation of artifacts that exhibit such behavior [3]. These processes require the training of models from data sources, in some cases heterogeneous. Training with a learning view involves the acquisition and pre-processing of information and the application or construction of rules for the treatment and use of this, to generate reasoning. That is, it uses the rules to reach approximate or definitive conclusions and self-correction [4].

Artificial Intelligence has become more popular today thanks to Big Data, advanced algorithms, and computers with improved power and storage, systems based on artificial intelligence are becoming an integrated element of digital systems and more specifically they are generating a profound impact on human decision-making. As a result, there is a growing demand for information systems researchers to investigate and understand the implications of this, for decision making and contribute to the theoretical advancement and practical success of the applications of this area of knowledge [5].

In 1959, Arthur Samuel coined the term Machine Learning and defined it as “the field of study that gives computers the ability to learn without being explicitly programmed”. Machine learning is part of the field of Artificial Intelligence, and its objective is usually to recognize and fit statistics to models [6]. Along with Artificial Intelligence, Machine Learning has emerged as the method of choice for the development of practical software for image and speech recognition, natural language processing, robot control, and other applications like Human Activity Recognition (HAR). Many developers of Artificial Intelligence systems recognize that, for many applications, it may be easier to train a system by feeding it examples of the desired input and output behavior, than to manually program in advance the desired response for all possible inputs [7].

Machine Learning has been playing, in recent decades, an important role in the construction of models based on experience from processed data [8], enabling computers to build models from data For example, according to the automation of decision-making processes, based on the input data [6], for their part [8] affirms that the study and construction of algorithms are explored who can learn and make predictions from data. This systematic review of the literature locates the advances made in Human Activity Recognition in each of the automatic learning methods, their evolution, and results.

The recognition of human activities has become one of the most used areas of knowledge that has allowed many advances in the care of patients at home and the improvement of the quality of life of the elderly. That is why different ways have been used by which the data from the different datasets can be processed, among which are machine learning and metaheuristics. The HAR approach is based on the complexity associated with the different data inputs that can come from wearable sensors, object sensors, images, audio, among others. Many models have been developed to try to improve performance and quality metrics based on different experimentations. Motivated by this research eld, the main contribution of this paper is (a) Show researchers the datasets most used in the literature for experimentation processes, as well as detailing the most used algorithms in their analysis. (b) Identify for each one of the data processing approaches the results of the experimentations of the algorithms, as well as discriminate the quality metrics associated with said applications an (c) Suggest, based on the analysis of the literature, the different techniques to be used to obtain good results in the experiments and show future researchers what the results of current applications are for the improvement of their experiments.

For the development of this purpose, a compilation of 570 articles has been analyzed, extracted from specialized databases such as IEEE Xplorer, Scopus, Science Direct, WOS. These manuscripts were analyzed through a meta-analytic matrix that allowed extracting relevant information such as year of publication, the database used, techniques implemented, results of the quality metrics implemented.

This article is a review of the literature on the use of the technique of the different machine learning methods supervised, unsupervised, ensembled, deep learning, reinforcement learning. First, a concept map of HAR Approach Concept Maps is shown (Section 2). Second, conceptual information is displayed (Section 3). Third, the methodology for analyzing the information sources is detailed (Section 4). Fifth, is the Scientometric Analysis (Section 5). Sixth, Technical Analysis (Section 6). Seventh, Conclusions (Section 7). Finally, the Future Works are shown (Section 8).

## 2. HAR Approach Concept Maps

**Figure 1:**
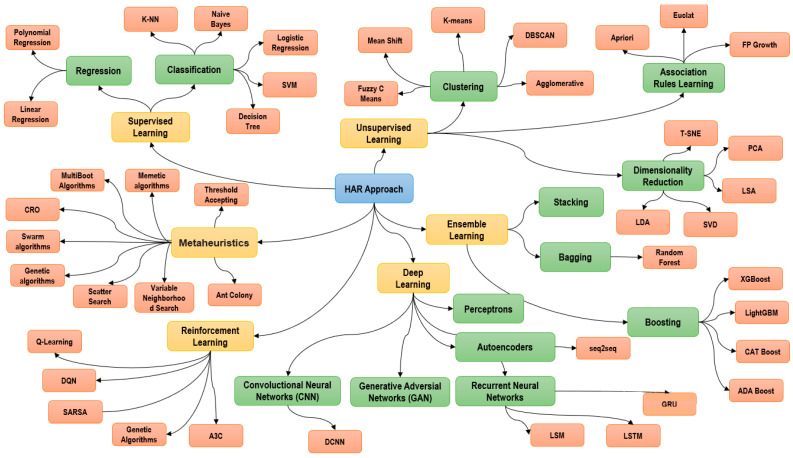
HAR Approach Concepts Maps.

In the last decades, Machine Learning has evolved in different methods and techniques to address different challenges in different areas of knowledge. In Figure 1 you can see the discrimination of each of the data mining methods can be seen. From classic supervised or unsupervised-based machine learning. Among the most outstanding algorithms of Supervised Analysis (Section 3.1), the following can be highlighted: Decision Tree [9], Support Vectorial Machine [10], Naive Bayesian Classifier [11], Artificial Neural Networks [12], Decision Tables [13] and Logistic Models [14], among others. Regarding Unsupervised Learning (Section 3.2), several methods can be found, among which Clustering [15] and Association Rules [16], and Dimensionality Reduction [17] can be highlighted. As for Ensembled Learning (Section 3.3), techniques such as Stacking [18], Bagging [19], Boosting [20] can be highlighted. Later, it was emphasized in methods or techniques based on Deep Neural Networks (Section 3.4) [21] that have several levels of analysis for knowledge discovery. Nowadays, machine learning has evolved to analysis based on Reinforcement Learning (Section 3.5) [22], which allows the algorithm that is strengthened in a system of rewards and punishments to permeate the learning process. In (Section 3.6) Metaheuristic Techniques [23], are strategies for designing heuristic procedures. Therefore, the types of metaheuristics are established, in the first place, based on the type of procedures to which they refer. The following can be identified among other types of algorithms: Threshold Accepting [24], Memetic algorithms [25], MultiBoot Algorithms [26], CRO (Coral reef-based algorithms) [27], Swarm algorithms [28], Genetic algorithms [29], Scatter Search [30], Variable Neighborhood Search [31] and Ant Colony [32]. Finally, in (Section 3.7), we show the approach of Transfer Learning [33] to Human Activity Recognition using a different type of combination of Neural networks for the analysis.

## 3. Conceptual Information

### 3.1. Supervised Learning

Supervised learning is a technique for deducing a function from training data. Training data consists of pairs of objects (usually vectors): one component of the pair is the input data and the other is the desired results. The output of the function can be a numeric value (as in regression problems) or a class label (as in classification problems). The goal of supervised learning is to create a function capable of predicting the value corresponding to any valid input object after viewing a series of examples, the training data. To do this, you must generalize from the data presented to previously unseen situations. Among the techniques most used in machine learning, the following can be highlighted.

#### 3.1.1. Decision Tree

According to Timaran [34], the quality of the decision tree depends on the size and precision of the classification. A subset of the dataset set (training) is chosen and a decision tree is created [9]. If it does not return the answer for the objects in the test set, a selection of exceptions is added to the dataset set, continuing the process until the correct decision set is found. The most used classification algorithms, from the decision trees category, are ID-3, C4.5, CART, Sprint, and j48 [34].

#### 3.1.2. Support Vectorial Machine (SVM)

In SVMs [10] the input quantities are mapped non-linearly to a very high-dimensional feature space. In this feature space, a line decision surface is constructed [35]. According to Hang [36], SVM uses a non-linear mapping to transform the original training data into a higher dimension. Within this new dimension, it looks for the linear optimal separation hyperplane (that is, a “decision limit” that separates the tuples of one class from another). SVMs can be used for numerical prediction, as well as for classification. They have been applied to several areas, including handwritten digit recognition, object recognition, and speaker identification, as well as benchmark time series prediction tests.

#### 3.1.3. Naïve Bayesian Classifier

Is a special typology of machine learning algorithms that address the task of classification [11]. The foundation of this is based on the “Bayes theorem”. In this algorithm, it is assumed that the variables that are used for the prediction are independent of each other. In other words, the presence of a series of characteristics in a data set is not related to the absence of another character in another data set.

#### 3.1.4. Artificial Neural Networks-ANN

The fundamental processing elements of an ANN are artificial neurons (or nodes) that are interconnected by weighted links that form layers [12]. Normally in an ANN, there is an input layer and an output layer, and several hidden layers that vary depending on the complexity of the problem in question. Neurons transform weighted input into output, using an activation function that can take different linear and non-linear forms. The process by which the weights are adjusted is called learning. Several non-linear ANNs are known to perform function approximators. Several parameters define the architecture of a neural network: the type of connection, learning rule, and activation functions. Due to these conformation parameters, there are different types of ANN, for example, Multilayer Perceptron—MLP [37], Echo State Networks-ESN, radial basis function-RBFN, Boltzmann machine.

#### 3.1.5. Decision Tables

The decision tables or also called decision rules achieve a synthetic representation of knowledge [13]. There are at least four sources of inconsistencies in decision tables, listed below: (1) hesitation in evaluating decision attribute values, (2) errors in recording, measurement, and observation, (3) condition attributes missing related to the evaluation of the decision attribute values, (4) the unstable nature of the system represented by the decision table and the like. These inconsistencies cannot be considered simple errors or noise. To acquire rules, from inconsistent decision tables, relative attribute reductions are needed. Skowron and Rauszer introduced the discernibility matrix method which became a popular approach for listing all reductions in the Rough set [26].

#### 3.1.6. Tree-Based on the Logistic Model-LMT

This classification process mixes decision trees with logistic regression [14]. The classification process can be improved if characteristics selection techniques are used, these allow assigning the prioritization or relevance of the attributes using the class criterion, thus obtaining a structure of attributes that directly affect the model and that in turn. are increasingly relevant concerning classification.

### 3.2. Unsupervised Learning

Unsupervised learning is a Machine Learning method where a model is fit for observations. It is distinguished from supervised learning by the fact that there is no a priori knowledge. In unsupervised learning, a data set of input objects is processed. Thus, unsupervised learning typically treats input objects as a set of random variables, with a density model being constructed for the data set. There are different unsupervised learning methods, among which we can highlight: Clustering, Association Rules, and Dimensionality Reduction.

#### 3.2.1. Clustering Methods

In the last decades, many are algorithms of grouping have been proposed and developed [38,39], from the hierarchical approach (Single Link, Complete Link, etc.) and partition (K-means, Gaussian Mixture, Density Estimation and Mode Seeking, etc.) among other methods. As data sets get larger and more varied, many of the dimensions are often irrelevant. These irrelevant dimensions can confuse traditional clustering algorithms.

Clustering is a technology used for many purposes because it simplifies massive data by extracting essential information, based on the relationship of subsequent analyzes or processes that make the process feasible or more efficient. For example, in information systems, grouping is applied to text documents or images to speed up indexing and retrieval [40,41]. Clustering can also be a stand-alone process and has been used as a technique for prototype-based supervised learning algorithms and different applications have also been made in non-vector data. The application of Clustering algorithms for the analysis of unsupervised data has become a useful tool to explore and solve the different application problems of data mining. Clustering methods [39,42] have been used to solve problems emanating from different contexts and disciplines, see Table 1.

**Table 1:** Clustering’s methods and applications.

Method	Strategy	Applications	
Hierarchical	Agglomerative	Nearest Neighbor [43] Farthest Neighbor [44] Average Linkage Pool [45] Minimum Variance [46] Median Method [47]	
	Divisive		
Non-Hierarchical	Reassignment	Centroids	K-means [48], QuickCluster [49], Forgy Methods [50]
		Medioid	k-medioids [51], Clara [52]
	Density	Dynamic clouds [53]	
		Typological approximation	Modal Analysis [54], Taxmap Method [55], Fortin Method [56]
		Probabilistic approximation	Wolf Methods [57]
	Direct	Block Clustering [58]	
	Reductive	Type Q Factor Analysis [59]	

#### 3.2.2. Association Rules Methods

The association rules base their analysis on the “if-then” algorithmic sentences, which allow supporting the different probabilities existing in the multiple elements of the data, found in large databases of different formats and types. The data mining techniques that are based on association rules throughout their evolution have had multiple applications, among which the sales and analysis of medical data sets can be highlighted.

Based on the algorithmic “if-then” sentences and based on established criteria such as support and trust, the association rules can identify the most important patterns. The support criterion gives the association rules the ability to know the frequency of the appearance of the elements in the data set is. As for the confidence criterion, it can determine the number of times the Boolean value of the “if-then” statement is true. There is also another common metric which is called Fit, which is fundamentally based on making a comparison between the expected confidence and the confidence that can be evidenced in the data. In the literature review, the progress of the association rules can be identified, as detailed below, see Table 2.

**Table 2:** Association Rules Evolutions.

Based in	Algorithms
Frequent Itemsets Mining	Apriori [60]
	Apriori-TID [61]
	ECLAT TID-list [62,63]
	FP-Growth [64]
Big Data Algorithms	R-Apriori [65]
	YAFIM [66]
	ParEclat [67]
	Par-FP (Parallel FP-Growth with Sampling) [68]
	HPA (Hash Partitioned Apriori) [69]
Distributed algorithms	PEAR (Parallel Efficient Association Rules) [70]
Distributed algorithms for fuzzy association rule mining	Count Distribution algorithm [71,72]

#### 3.2.3. Dimensionality Reduction Methods

Dimensionality reduction methods are statistical techniques that map the data set to subspaces derived from the original space, of less dimension, which allow a description of the data at a lower cost. These techniques become important as many algorithms from various fields such as numerical analysis, machine learning or data mining tend to degrade their performance when used with high dimensional data. In external cases, the algorithm is no longer useful for the purpose for which it was designed. The curse of dimension refers to the various phenomena that arise when analyzing and organizing data from multi-dimensional spaces. Among the most important algorithms we can highlight.

Missing Values Ratio [73]: By examining the data, if we find that it contains many missing values, if there are few missing values, we can fill in the missing values or remove this variable directly; when the proportion of missing values in the dataset is too high, I usually choose directly Remove this variable because it contains too little information. Specific removal is not removed, how to remove depends on the situation, we can set a threshold, if the proportion of missing values is greater than the threshold, remove the column where it is. The higher the threshold. The more aggressive the dimensionality reduction method.

Low Variance Filter [74]: If the value of a column is the same in a dataset, that is, its variance is very low, we generally think that low-variance variables contain very little information, so you can eliminate it directly and put it into practice, which is to calculate all Variation Size variables and then eliminate the smallest of them.

High Correlation Filter [75]: If the two variables are highly correlated, this means that they have similar trends and can carry similar information. Similarly, the presence of such variables can reduce the performance of certain models (such as linear and logistic regression models). To solve such problems, we can calculate the correlation between independent variables. If the correlation coefficient exceeds a certain threshold, one of the variables is eliminated.

Random Forests/Ensemble Trees [76]: Random Forest is a widely used feature selection algorithm, it automatically calculates the importance of each feature, so no separate programming is required. This helps us choose a smaller subset of features. The advantages of the random forest: high precision, the introduction of randomness makes random forests not easy to overfit, the introduction of randomness makes the random forests have good anti-noise ability (can better handle outliers), can handle very high-dimensional data without feature selection, it can handle both discrete data and continuous data, and the data set does not need to be normalized, fast training speed, you can get the importance of variable classification and easy to parallelize. Disadvantages of the random forest: when there are many decision trees in the random forest, the space and time required for training will be large, and the interpretability of the random forest is poor.

Principal Component Analysis (PCA) [77]: PCA is a very common dimensionality reduction method. You can reduce the number of predictors by reducing the dimensionality of high-dimensional data while eliminating noise through dimensionality reduction. The most direct application is to compress data, mainly used in signal processing Noise reduction, and visualization after data dimensionality reduction.

### 3.3. Ensemble Learning

An ensemble is a set of machine learning models. Each model produces a different prediction. The predictions from the different models are combined to obtain a single prediction. The advantage we get from combining different models is that because each model works differently, its errors tend to be compensated for. This results in a better generalization error.

#### 3.3.1. Voting by the Majority

Training multiple machine learning models with the same data [78]. When we have new data, we will get a prediction from each model. Each model will have a vote associated with it. In this way, we will propose as a final prediction what most of the models vote for. There is another way to combine voting. When machine learning models give a probability, we can use “soft-voting”. In soft voting, more importance is given to results in which some model is very confident. That is, when the prediction is very close to probability 0 or 1, more weight is given to the prediction of that model.

#### 3.3.2. Bagging

Unlike majority voting, the way to get errors to compensate for each other is that each model is trained with subsets of the training set [79]. These subsets are formed by randomly choosing samples (with repetition) from the training set. The results are combined, for classification problems, as we have seen in majority voting, with the soft vote for the models that give probabilities. For regression problems, the arithmetic mean is normally used.

#### 3.3.3. Boosting

In boosting, each model tries to fix the errors of the previous models [80]. For example, in the case of classification, the first model will try to learn the relationship between the input attributes and the result. You will surely make some mistakes. So, the second model will try to reduce these errors. This is achieved by giving more weight to poorly classified samples and less weight to well-classified samples. For regression problems, predictions with a higher mean square error will have more weight for the next model.

#### 3.3.4. Stacking

When we talk about a stacking ensemble, we mean that we are stacking models [81]. When we stack models, what we are doing is using the output of multiple models as the input of multiple models.

### 3.4. Deep Learning

Deep Learning is a type of machine learning that is structured and inspired by the human brain and its neural networks [82]. Deep learning processes data to detect objects, recognize conversations, translate languages, and make decisions. Being a type of machine learning, this technology helps artificial intelligence learn continuously. Deep learning is based on the use of artificial neural networks. Within neural networks 3 types are the most used.

#### 3.4.1. Convolutional Neural Networks (CNN)

Convolutional neural networks are artificial neural networks that have been designed to process structured matrices, such as images [83]. That is, they are responsible for classifying images based on the patterns and objects that appear in them, for example, lines, circles, or even eyes and faces.

#### 3.4.2. Recurrent Neural Networks (RNN)

Recurrent neural networks are neural networks that use sequential data or time-series data [84]. These types of networks solve ordinal or temporal problems, such as language translation, speech recognition, Natural Language Processing (NLP, Natural Language Processing), and image capture. Therefore, these networks are in technologies such as Siri or Google translate. In this case, natural language processing recognizes a person’s speech. For example, it is distinguished if the person who is speaking is a man or a woman, an adult or a minor, if they have an Andalusian or Catalan accent, etc. In this way, the person’s way of speaking is analyzed, and their idiolect is reached.

#### 3.4.3. Generative Adversarial Networks (GAN)

The antagonistic generative networks consist of using 2 artificial neural networks and opposing them to each other (that is why they are known as antagonistic) to generate new content or synthetic data that can be passed as real [85]. One of the networks generates and the other works as a “discriminator”. The discriminatory network (also known as an antagonistic network) has been trained to recognize real content and acts as a sensor for the network that generates content to make content that appears real.

### 3.5. Reinforcement Learning

The field of machine learning is the branch of Artificial Intelligence that encompasses techniques that allow machines to learn through their environment. This environment can be considered as the set of data that the algorithm has or obtains in the training stage. Reinforcement learning is the most common in nature. An individual has a connection with the environment with which he obtains information from the cause-effect relationships, the results of the actions carried out, and the strategy to follow to complete an objective [86].

The time difference method was introduced by Sutton [87] as a model-free method based on a bootstrapping update rule and consists of estimating the values of immediate and future rewards in a way like programming. Are dynamic and are denoted as TD (λ). Methods of time difference attempt to estimate the value function of a given state of a policy, and contrary to Monte Carlo methods, you do not need to wait at the end of an episode to make such an estimate. Some prominent algorithms are.

#### 3.5.1. SARSA

One of the algorithms that derive from the method based on time difference is the SARSA algorithm [88] which is an on-policy method, that is, it has an initial policy and updates it at the end. of each episode.

#### 3.5.2. Q-Learning

Q-learning is a value-based learning algorithm that focuses on optimizing the value function according to the environment or problem [89,90]. The Q in Q-learning represents the quality with which the model finds its next quality-improving action. The process can be automatic and simple. This technique is great to start your reinforcement learning journey. The model stores all the values in a table, which is Table Q. In simple words, the learning method is used for the best solution.

#### 3.5.3. Deep Reinforcement Learning

Deep Reinforcement Learning [91], where reinforcement learning is integrated with neural networks. The DeepMind company began to use this type of learning to create agents that would learn to play Atari games from scratch without having any information about them, not even the rules of the video game.

### 3.6. Metaheuristic Learning

Metaheuristics are clever strategies to design or improve very general heuristic procedures with high performance. The term metaheuristic first appeared in Fred Glover’s seminal article on tabu search in 1986 [92]. Since then, many proposals for guidelines have emerged to design good procedures to solve certain problems that, by expanding their field of application, have adopted the denomination of metaheuristics.

Some of the main types are: Relaxation metaheuristics [93] refer to problem-solving procedures that use relaxations of the original model (that is, modifications of the model that make the problem easier to solve), the solution of which facilitates the solution of the original problem. The constructive metaheuristics [94] are oriented to the procedures that try to obtain a solution from the analysis and gradual selection of the components that form it. Search metaheuristics [95] guide procedures that use transformations or moves to traverse the space of alternative solutions and exploit the associated environment structures. Evolutionary metaheuristics [96] are focused on procedures based on solution sets that evolve over the solution space.

### 3.7. Transfer Learning

Deep Learning primarily emphasizes features, Reinforcement Learning primarily emphasizes feedback, and Transfer Learning primarily emphasizes adaptation. Traditional machine learning is about reaping the benefits of planting fruits and reaping the benefits of planting beans, while transfer learning can draw inferences from each other.

Artificial intelligence competition, from algorithm model development to data quality and data competition, these successful models and algorithms are mainly driven by supervised learning, and supervised learning consumes a lot of data and requires big data support (big data) to meet the precise requirements of the application. The development of artificial intelligence tends to satisfy the precise requirements of the applications without requiring massive data. Therefore, “small data learning” is becoming a new point of interest. Small data learning techniques represented by migration learning and reinforcement learning can better reflect artificial intelligence.

Since the transfer learning (TL) concept was proposed by Stevo Bozinovski and Ante Fulgosi in 1976 [97], it has received a great deal of attention from the academic community. The definition of transfer learning is too broad and a variety of specialized terms have appeared in related research, such as learning to learn, lifelong learning, multitasking learning, meta-learning, inductive transfer, knowledge transfer, context-sensitive learning, etc. Among them, transfer learning has the closest relationship with multitasking learning. Multitask learning learns multiple different tasks at the same time and discovers implicit common features to aid single-task learning.

### 3.8. Human Activity Recognition

Recognizing human activities consists of interpreting human gestures or movements through sensors to determine human action or activity [98]. For example, a HAR system can report activities performed by patients outside of hospital facilities, which makes it a useful tool for evaluating health interventions and therapy progress, and for clinical decision-making [99]. HAR can be supervised or unsupervised. The supervised HAR system requires prior training with a tagged data set, on the contrary, the unsupervised system does not require training but has a set of rules configured during development. In this particular work, we focused on a HAR system of the supervised type to recognize the following six human activities: walking (WK), climbing stairs (WU), descending stairs (WD), standing (ST), lying down (LD), and being sitting (SD). We name, in particular, the WK, WU, and WD activities as dynamic activities since they involve a voluntary movement that causes displacement and is reflected in the inertial sensors, and we call ST, LD, and SD activities. Given that they do not involve voluntary movements of the subject and there is no displacement of the person.

In HAR systems it is common to use signals and images that come from sensors that can be located in a specific physical space, such as in a room, or that can be placed or carried by people, like those found in smart cell phones or smartwatches. Smartphones are mobile phones that can perform tasks like those of a computer, such as the ability to store and process data and be able to navigate the Internet [100]. In addition, compared to personal computers, smartphones are widely accepted due to their small size, low weight, more personal device, and, especially, great connectivity that allows you to access at any time and place to information sites and social networks [101]. Other applications that are usually present are integrated cameras, contact management, multimedia software capable of playing music and being able to view photos and videos, and the use of navigation programs, and, in addition, having the ability to view business documents in different formats such as PDF and Microsoft Office [101].

**Figure 2:**
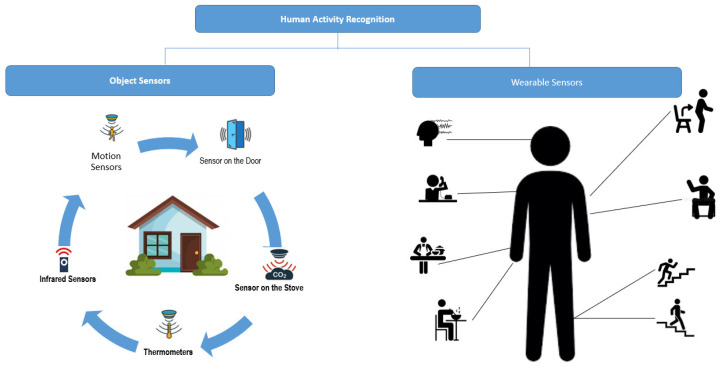
Sensors of Human Activity Recognition.

Currently, different sensors are installed, such as positioning sensors, proximity sensors, temperature sensors, accelerometer, gyroscope, magnetometer, microphone, etc., as shown in Figure 2. This is currently a challenge carried out by different scientific communities, particularly in the fields of computer vision, signal processing, and machine learning. The sensors are usually operated by a microcontroller or microprocessor, which performs the function of a computer.

Inertial sensors are sensors based on the principle of inertia, the tendency of a body to conserve its speed (in the absence of an external influence, a body remains in a uniform rectilinear motion). There are different types of sensors to measure signals that can be used by HAR systems. Two of the most used are the accelerometer and the gyroscope. The accelerometer measures the acceleration (in meters per second squared, m/s^2^) based on the different variations that a capacitance makes inside the sensor. This capacitance is a microelectromechanical system (MEMS for its acronym in English microelectromechanical systems) that consists of the suspension of silicon particles that are located at a fixed point and are moved freely in the axis where they are measured. When acceleration occurs, the particles move and break with equilibrium in capacitance; this is measured to provide the information that occurs in a certain axis.

According to the type of sensors and the occupation of the indoor environments, a series of datasets have been built that have served to carry out different experiments based on machine learning techniques. The most prominent datasets are: UCI HAR [102], KU-HAR [103], Precis HAR [104], Fall-Up Dataset [105], VanKasteren [106], CASAS Multiresident [107], WISDM [108], DOMUS [109], Opportunity [110], CASAS Aruba [111], USC-HAD [112], MIT PlaceLab [113], Smart Environment—Ulster University [114], CASAS–Daily Life Kyoto [115], PAMAP2 [116], mHealth [117], DSADS [118], UJAmI SmatLab [119].

## 4. Methodology for Analyzing the Information

The methodology for the analysis of the publications is supported and defined by Kitchenham [120]. This methodology consists of identifying the main research problem, and then disaggregating each of its components by analyzing the different inclusions and exclusions, to determine a suitable search string to be used in scientific databases. Specifically for our case study, in addition to the Scientometric type variables, those related to the type of dataset used, techniques or algorithms implemented, as well as the quality of the results measured by the quality metrics, were identified. Kitchenham [120] defines different stages of the literature review process, among which the following can be highlighted: (a) Identification of the search parameters (search objectives, hypotheses identified) (b) Definition of search engines (selection of specialized databases where the study is to be developed) (c) Response to the hypotheses that were raised for the literature inquiry process.

**Figure 3:**
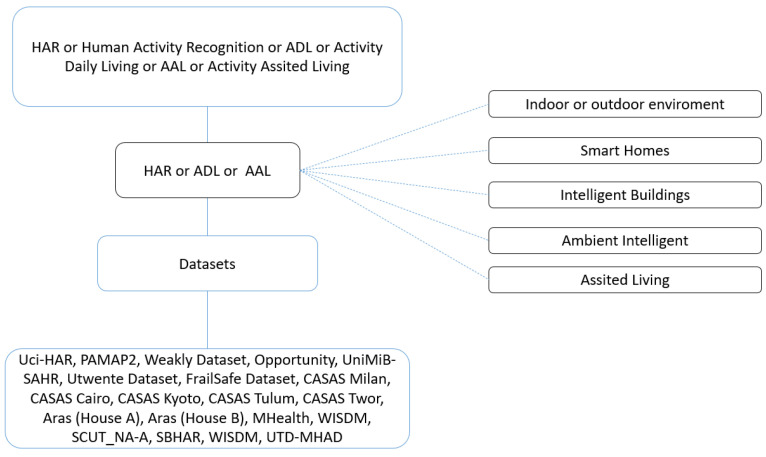
Relationship between concepts for the literature review.

By these previously defined phases, the first thing to do is to identify the central question of the inquiry process. For this literature review, it would be “What are the different techniques based on Machine Learning that support the analysis of dataset recognition of human activities?”. To carry out the literature review, the IEEE, Scopus, Science Direct, and WOS databases were used. To delimit the documentary findings, the following search string was used: (HAR OR ADL OR AAL) AND dataset AND (“indoor environment’’ OR “smart homes” OR “intelligent buildings” OR “ambient intelligence” OR “assisted living’’). In Figure 3 you can see the basic concept scheme for the review document filter. Then the references were analyzed by the machine learning technique that is implemented, which is described in Section 6.

It is important to specify that the order of the different terms that are observed in Figure 3 determine all those that are part of the domain of knowledge, which was previously tested in the different search engines of the scientific databases to eliminate the different noises from them. that can be generated at the time of the search and the exclusion of papers not related to the study area. Taking into account the previously explained methodology, different factors of the analytical order and high importance for those interested in this area of knowledge were described in the meta-analytic matrix, such as year of publication of the work (which is not greater than a window of 5 years), journal, conference or book where the publication was made, quartile in the case of publications in journals, country of origin of the first author as well as the university or research center. Other technical variables are taken into account in the same way for the development of this research, such as Name of the dataset, type of data collection, type of activities carried out, several individuals who define the occupation, data mining techniques used, hybridization of techniques, results of quality metrics.

## 5. Scientometric Analysis

**Figure 4:**
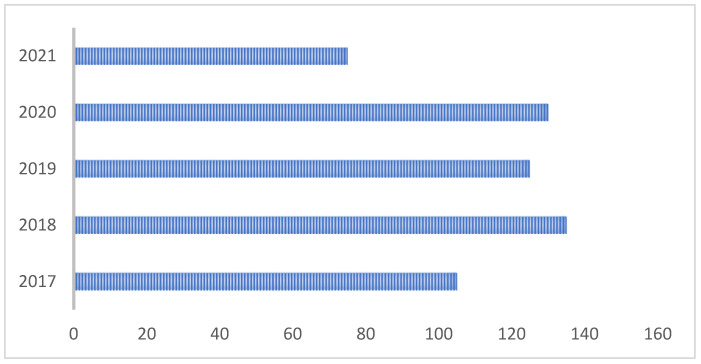
Years of publication of the articles.

In the results obtained from the 570 articles processed, different relevant variables were taken into account, among which are detailed: (1) the year of publication of the article see Figure 4, (2) the database where the publication can be found, (3) Type of publication if it is a journal, conference or book, (4) Quartile of the journal in the case of publications in this medium, (5) country of origin of the journal (6) country of origin of the first author of the article (7) University of the first author, (8) Dataset used for the experiments, (9) Techniques used for the discovery of information and (10) results of the metrics of each technique.

**Figure 5:**
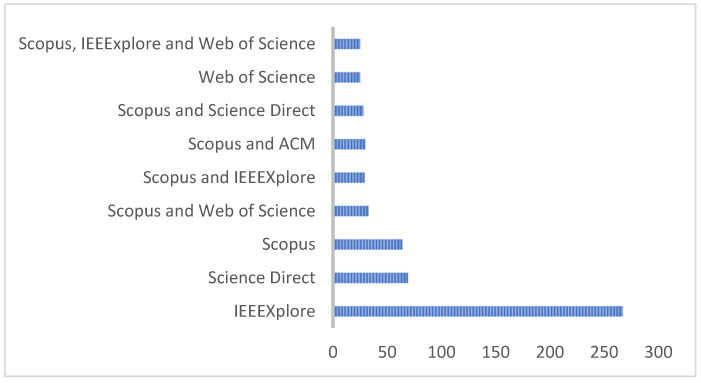
Years of publication of the articles.

It can be identified that 2018 was where the most publications were generated in HAR’s line of work. In the same way, when discriminating the databases in which the publications are made, it is highlighted that most of the works have been published in the Science Direct database, then Scopus. Some publications are visible in different databases, as shown in Figure 5.

**Figure 6:**
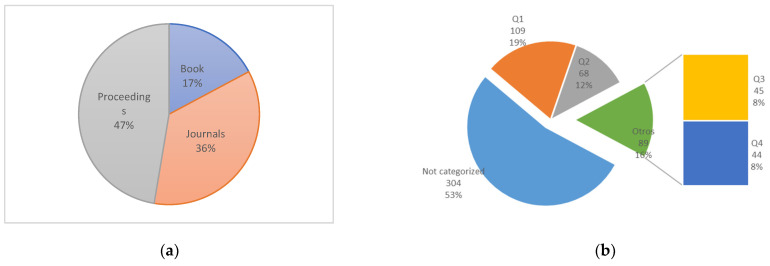
(**a**) Publications division according to typology (**b**). Distribution by quartiles of publications.

Of the total articles, analyzed 64% of them refer to conference publications, 4 & are books and 36% refer to journals, see Figure 6a,b.

## 6. Technical Analysis

### 6.1. Supervised Learning Applied to Human Activity Recognition Dataset

Regarding the application of Machine Learning techniques, to Human Activity Recognition Dataset, various experiments have been developed, but the most relevant ones found in the literature are highlighted below (see Table 3). Tasmin [121], carried out implementations in the UCI-HAR Dataset, through the implementation of supervised algorithms Nearest Neighbor, Decision Tree, Random Forest, and Naive Bayes, of the techniques used, the one with the best results in the detection of activities was the Bayesian with an accuracy of 76.9%. Igwe [122], concentrated his experimentations on the ARAS Data-set which was implemented in 2 different locations (House A and House B), CA-SAS Tulum created by WSU University, the author applied supervised techniques such as SVM, ANN, and MSA (Margin Setting Algorithm), demonstrating the effectiveness of the latter in identifying activities with an accuracy of 68.85%, 96.24% and 68% in the respective Datasets.

Subasi [123], performed analysis on the Meath Dataset, applying techniques such as K-NN, ANN, SVM, C4.5, CART. Random Forest and Rotation Forest obtained better results with SVM and Random Forest with 99.89%. Maswadi [124], firstly I prepare the Dataset using Sliding window segmentation techniques with a variable size in different Datasets such as WISDM with SCUT_NA-A, SCUT NA-.An only, PAMPA2 with Mhealth, SBHAR, WISDM, UTD-MHAD, Groupware, Free-living WISDM with Skoda, UniMiB SHAR, and Groupware, showing the superiority of this technique obtaining results greater than 80% accuracy. Other authors such as Damodaran [125], applied SVM, LSTM to the CSI-Data Dataset, where better results are shown in the use of SVM with 96%.

Other authors such as Saha [126] and Das [127], define the characteristics and process for the construction of their Dataset, to which a set of techniques are applied and it should be noted that both authors show that vector support machines show efficiency in the results of classification of human activities. Franco [128], uses techniques such as FFNN, SVM, and LSTM in the UK-Dale Dataset, showing the effectiveness of FFNN with 95.28% accuracy in quality metrics.

Bozkurt [129] and Wang [130], carry out supervised learning implementations in the UCI HAR Dataset, with various combined supervised techniques, and in the case of Bozkurt, they describe that using SVM + KNN obtains good results in the classification with an accuracy of 96.71% and Wang explains that using a combination of Decision Tree it is possible to count on the accuracy of 92.73%. Outreach [131], performs analysis on two Datasets of the set CASAS Tulum and Two, highlighting the use of BackPropagation with results 88.75% and 76.9%, respectively in accuracy.

Demrozi [132], performs multiple experiments of many supervised techniques in many widely known Datasets such as WISDM, DAPHNET, PAPAM, HHAR (Phone), HHAR (watch), Mhealth, RSSI, CSI. For this, different algorithms are implemented such as KNN, LDA, QDA, RF, DT, CNN. In the case of the algorithm WISDM, DAPHNET, PAPAM, HHAR (Phone), HHAR (watch) the RF algorithm obtains the best results with accuracy of 90%, 91%, 80%, 88%, 85% precision, and recall of 91%, 91%, 83%, 89%, 85%. For Mhealth, RSSI, the performance of the QDA algorithm is denoted with 91% and 92% in accuracy and 85% and 92% in precision and recall respectively.

Xu [133], applies compares techniques such as DT, SVM, KNN, AdaBoost, DCNN in Dataset CASAS Aruba, showing the superiority of ensembled techniques such as Adaboost with the accuracy of 98%, precision 96%, recall 95.9%, and f -measure 95.9%. Other authors such as Hussain [134] apply algorithms such as SVM, Random Forest, KNN to datasets such as SisFall, the SVM results being better with 97.77% accuracy. Finally, Liciotti [135], performs experimentation on a set of well-known CASAS project Datasets such as Milan, Cairo, Kyoto 2, Kyoto 3, Kyoto 4, of algorithms such as Naive Bayes, HMM + SVM, CRF, LSTM, showing the superiority of LSTM in the results.

**Table 3:** Supervised Techniques results.

Dataset	Technique	Metrics				References
		Accuracy	Precision	Recall	F-Measure	
UCI Machine Learning	Nearest Neighbor	75.7	-	-	-	[121]
	Decision Tree	76.3	-	-	-	
	Random Forest	75.9	-	-	-	
	Naive Bayes	76.9	-	-	-	
Aras (House A)	MSA (Margin Setting Algorithm)	68.85	-	-	-	[122]
	SVM	66.90	-	-	-	
	ANN	67.32	-	-	-	
Aras (House B)	MSA (Margin Setting Algorithm)	96.24	-	-	-	
	SVM	94.81	-	-	-	
	ANN	95.42	-	-	-	
CASAS Tulum	MSA (Margin Setting Algorithm)	68.00	-	-	-	
	SVM	66.6	-	-	-	
	ANN	67.37	-	-	-	
Mhealth	K-NN	99.64	-	-	99.7	[123]
	ANN	99.55	-	-	99.6	
	SVM	99.89	-	-	100	
	C4.5	99.32	-	-	99.3	
	CART	99.13	-	-	99.7	
	Random Forest	99.89	-	-	99.89	
	Rotation Forest	99.79	-	-	99.79	
WISDM, SCUT_NA-A	Sliding window with variable size, S transform, and regularization based robust subspace (SRRS) for selection and SVM for Classification	96.1	-	-	-	[124]
SCUT NA-A	Sliding window with fixed samples, SVM like a classifier, cross-validation	91.21	-	-	-	
PAMPA2, Mhealth	Sliding windows with fixed 2s, SVM, and Cross-validation	84.10	-	-	-	
SBHAR	Sliding windows with fixed 4s, SVM, and Cross-validation	93.4	-	-	-	
WISDM	MLP based on voting techniques with nb-Tree are used	96.35	-	-	-	
UTD-MHAD	Feature level fusion approach& collaborative representation classifier	79.1	-	-	-	
Groupware	Mark Hall’s feature selection and Decision Tree	99.4	-	-	-	
Free-living	k-NN and Decision Tree	95	-	-	-	
WISDM, Skoda	Hybrid Localizing learning (k-NN-LSS-VM)	81	-	-	-	
UniMiB SHAR	LSTM and Deep Q-Learning	95	-	-	-	
Groupware	Sliding windows Gaussian Linear Filter and NB classifier	89.5	-	-	-	
Groupware	Sliding windows Gaussian Linear Filter and Decision Tree classifier	99.99	-	-	-	
CSI-data	SVM	96	-	-	-	[125]
	LSTM	89	-	-	-	
Built by the authors	IBK	95	-	-	-	[126]
	Classifier based ensemble	98	-	-	-	
	Bayesian network	63	-	-	-	
Built by the authors	Decision Tree	91.08	-	-	89.75	[127]
	Random Forest	91.25	-	-	90.02	
	Gradient Boosting	97.59	-	-	97.4	
	KNN	93.76	-	-	93.21	
	Naive Bayes	88.57	-	-	88.07	
	SVM	92.7	-	-	91.53	
	XGBoost	96.93	-	-	96.63	
UK-DALE	FFNN	95.28	-	-	-	[128]
	SVM	93.84	-	-	-	
	LSTM	83.07	-	-	-	
UCI Machine Learning	KNN	90.74	91.15	90.28	90.45	[129]
	SVM	96.27	96.43	96.14	96.23	
	HMM+SVM	96.57	96.74	96.49	96.56	
	SVM+KNN	96.71	96.75	96.69	96.71	
	Naive Bayes	77.03	79.25	76.91	76.72	
	Logistic Reg	95.93	96.13	95.84	95.92	
	Decision Tree	87.34	87.39	86.95	86.99	
	Random Forest	92.3	92.4	92.03	92.14	
	MLP	95.25	95.49	95.13	95.25	
	DNN	96.81	96.95	96.77	96.83	
	LSTM	91.08	91.38	91.24	91.13	
	CNN+LSTM	93.08	93.17	93.10	93.07	
	CNN+BiLSTM	95.42	96.58	95.26	95.36	
	Inception+ResNet	95.76	96.06	95.63	95.75	
UCI Machine Learning	NB-NB	73.68	-	-	46.9	[130]
	NB-KNN	85.58	-	-	61.08	
	NB-DT	89.93	-	-	69.75	
	NB-SVM	79.97	-	-	53.69	
	KNN-NB	74.93	-	-	45	
	KNN-KNN	79.3	-	-	49.82	
	KNN-DT	87.01	-	-	60.98	
	KNN-SVM	82.24	-	-	53.1	
	DT-NB	84.72	-	-	60.05	
	DT-KNN	91.55	-	-	73.11	
	DT-DT	92.73	-	-	75.97	
	DT-SVM	93.23	-	-	77.35	
	SVM-NB	30.40	-	-	-	
	SVM-KNN	25.23	-	-	-	
	SVM-DT	92.43	-	-	75.31	
	SVM-SVM	43.32	-	-	-	
CASAS Tulum	Back-Propagation	88.75	-	-	-	[131]
	SVM	87.42	-	-	-	
	DBM	90.23	-	-	-	
CASAS Twor	Back-Propagation	76.9	-	-	-	
	SVM	73.52	-	-	-	
	DBM	78.49	-	-	-	
WISDM	KNN	69	78	-	78	[132]
	LDA	40	34	-	34	
	QDA	65	58	-	58	
	RF	90	91	-	91	
	DT	77	77	-	77	
	CNN	66	62	-	60	
DAPHNET	KNN	90	87	-	88	
	LDA	91	83	-	83	
	QDA	91	82	-	82	
	RF	91	91	-	91	
	DT	91	83	-	83	
	CNN	90	87	-	87	
PAPAM	KNN	65	66	-	66	
	LDA	45	45	-	45	
	QDA	15	19	-	19	
	RF	80	83	-	83	
	DT	60	60	-	60	
	CNN	73	76	-	73	
HHAR(Phone)	KNN	83	85	-	85	
	LDA	43	45	-	45	
	QDA	40	50	-	50	
	RF	88	89	-	89	
	DT	67	66	-	66	
	CNN	84	84	-	84	
HHAR(watch)	KNN	78	82	-	82	
	LDA	54	52	-	52	
	QDA	26	27	-	27	
	RF	85	85	-	85	
	DT	69	69	-	69	
	CNN	83	83	-	83	
Mhealth	KNN	76	81	-	81	
	LDA	38	59	-	59	
	QDA	91	82	-	82	
	RF	85	85	-	85	
	DT	77	77	-	77	
	CNN	80	80	-	80	
RSSI	KNN	91	91	-	91	
	LDA	91	91	-	91	
	QDA	91	91	-	91	
	RF	91	91	-	91	
	DT	91	91	-	91	
	CNN	91	90	-	91	
CSI	KNN	93	93	-	93	
	LDA	93	93	-	93	
	QDA	92	92	-	92	
	RF	93	93	-	93	
	DT	93	93	-	93	
	CNN	92	92	-	92	
Casas Aruba	DT	96.3	93.8	92.3	93	[133]
	SVM	88.2	88.3	87.8	88.1	
	KNN	89.2	87.8	85.9	86.8	
	AdaBoost	98	96	95.9	95.9	
	DCNN	95.6	93.9	95.3	94.6	
SisFall	SVM	97.77	76.17		75.6	[134]
	Random Forest	96.82	79.99		79.95	
	KNN	96.71	93.99		68.36	
CASAS Milan	Naive Bayes	76.65				[135]
	HMM+SVM	77.44				
	CRF	61.01				
	LSTM	93.42				
CASAS Cairo	Naive Bayes	82.79				
	HMM+SVM	82.41				
	CRF	68.07				
	LSTM	83.75				
CASAS Kyoto 2	Naive Bayes	63.98				
	HMM+SVM	65.79				
	CRF	66.20				
	LSTM	69.76				
CASAS Kyoto 3	Naive Bayes	77.5				
	HMM+SVM	81.67				
	CRF	87.33				
	LSTM	88.71				
CASAS Kyoto 4	Naive Bayes	63.27				
	HMM+SVM	60.9				
	CRF	58.41				
	LSTM	85.57				

### 6.2. Unsupervised Learning Applied to Human Activity Recognition Dataset

In the unsupervised learning applications in the literature, different applications of the algorithms can be observed that are measured with quality metrics associated with the groupings such as ARI, Jaccard Index, Silhouette Index, Euclidean, F1 Fisher’s discriminant (see Table 4). The following works developed by authors such as Wang [130] stand out, who uses various versions of the UCI-HAR Dataset, implementing algorithms such as K-means, HAC, FCM, both showing better results for the case of FCM. Mohmed [136] applies unsupervised algorithms like FCM to the Nottingham Trent University Dataset. Brena [137], applies his form developed by the author called PM Mo-del to perform unsupervised analysis to the Chest Sensor Dataset, Wrist Sensor Dataset, WISDM Dataset, and Smartphone Dataset, which he measures using the silhouette index. He [138], applies another method developed by the authors called wavelet tensor fuzzy clustering scheme (WTFCS) to the DSAD Dataset, obtaining an ARI index of 89.66%.

Wang [139], implements clustering-based algorithms such as Spectral Clustering, Single Linkage, Ward Linkage, Average Linkage, K-medioids to the UCI-HAR dataset, analyzing their Jaccard and Euclidean indices as shown in Table 4. In the same way, Bota [140] also makes experiments in the UCI-HAR and CADL Dataset with the K-means, K-Means 5, Spectral Clustering, Gaussian Mixture, DBSCAN algorithms analyzing its F1 Fisher’s discriminant rat.

**Table 4:** Unsupervised Techniques results.

Dataset	Technique	Metrics					References
		ARI	Jaccard Index	Silhouette Index	Euclidean	F1 Fisher’s Discriminant Ratio	
UCI HAR SmartPhone	K-means	0.7727	0.3246	0.4416			[130]
	HAC	0.4213	0.2224	0.5675			
	FCM	0.8343	0.4052	0.4281			
UCI HAR Single Chest-Mounted Accelerometer	K-means	0.8850	0.6544	0.6935			
	HAC	0.5996	0.2563	0.6851			
	FCM	0.9189	0.7230	0.7751			
Nottingham Trent University	FCM	-	-	-	-		[136]
Chest Sensor Dataset	PM Model			25.8%	-		[137]
Wrist Sensor Dataset				64.3%	-		
WISDM Dataset				54%	-		
Smartphone Dataset				85%	-		
DSAD	wavelet tensor fuzzy clustering scheme (WTFCS)	0.8966	-	-	-		[138]
UCI HAR	Spectral Clustering		0.543		0.583		[139]
	Single Linkage		0.807		0.851		
	Ward Linkage		0.770		0.810		
	Average Linkage		0.790		0.871		
	K-medioids		0.653		0.654		
UCI HAR	K-means					52.1	[140]
	K-Means 5					50.7	
	Spectral Clustering					57.8	
	Gaussian Mixture					49.8	
	DBSCAN					16.4	
CADL	K-means					50.9	
	K-Means 5					50.5	
	Spectral Clustering					61.9	
	Gaussian Mixture					58.9	
	DBSCAN					13.9	

### 6.3. Ensemble Learning Applied to Human Activity Recognition Dataset

In the lessons based on ensemble learning, the application of multiple techniques is usually carried out, which together offer better results (see Table 5). Below is a detailed description of the works found in the literature review that shows the application of these techniques in the recognition of human activities. Yacchirema [141], uses a combination of techniques such as Decision Tree, Ensemble, Logistic Regression, Deepnet to analyze the SisFall Dataset, explaining the results of the DeepNet algorithm with an accuracy of 99.06%. For his part, Manzi [142], uses a mixture of X-means and SV; to analyze the Cornell Activity Dataset and TST Dataset obtaining 98.4% and 92.7% respectively.

Ma [143], uses the model based on Multi-task deep clustering in the HHAR, MobiAct, MobiSense datasets, where the latter algorithm obtains an accuracy of 72.5%, a precision of 71.2%, and a recall of 70.7%. Budisteanu [144], describes the NTU-RGB + D Dataset, and implements the K-Means, GMM algorithms, obtaining 85.72% and 87.26% respectively. Xu [145], uses the well-known UCI-HAR Dataset, implementing the CELearning own technique, obtaining an accuracy of 96.88%.

Choudhury [146], also analyzes the UCI-HAR Dataset, with the algorithms RF, XGB, AdaB, GB, ANN, V. RNN, LSTM, DT, KNN, and NB, where the RF algorithm performs the best result in the ensemble models with 96.96%. Wang [147] for his part defines his Dataset to which he implements the algorithms GB, RFs, Bagging, XGB, AdaBoost, DT, MLP, LSVM, NLSVM, LR, KNNs, GNB, in which the RF algorithm obtains the best results with an accuracy of 83.9%. Jethanandani [148], works with the popular Dataset House A and House B, applying algorithms such as Bernoulli NB, Decision Tree, Logistic Regression, KNN. This experimentation shows the good results of the algorithms based on decision trees with 88% and 97.2% respectively.

Subasi [149], also uses the UCI-HAR Dataset, applying the algorithms SVM-AdaBoost, k-NN-AdaBoost, ANN-AdaBoost, NB-AdaBoost, RF-AdaBoost, CART-AdaBoost, C4.5-AdaBoost, REPTree-AdaBoost, LADTree-AdaBoost obtaining better results with the REPTree-AdaBoost combination with 99.95% accuracy. Padmaja [150], uses HAR Dataset, HAPT Dataset implementing the KN, CART, BAYES, RF, ET alalgorithms, a method proposed by the authors, demonstrating the superiority of the results of the method proposed by the authors.

**Table 5:** Ensembled Learning Techniques results.

Dataset	Technique	Metrics				References
		Accuracy	Precision	Recall	F-Measure	
SisFall	Decision Tree	97.48	-	-	-	[141]
	Ensemble	99.51	-	-	-	
	Logistic Regression	84.87	-	-	-	
	Deepnet	99.06	-	-	-	
Cornell Activity Dataset	X-means-SVM	98.4	95.0	95.8	-	[142]
TST Dataset		92.7	95.6	91.1	-	
HHAR	Multi-task deep clustering		67.2	65.3	65.9	[143]
MobiAct			68.3	69.1	66.8	
MobiSense			72.5	71.2	70.7	
NTU-RGB + D	K-Means	85.72	-	-	-	[144]
	GMM	87.26	-	-	-	
UCI HAR	CELearning	96.88%	-	-	-	[145]
UCI HAR	RF	96.96	97.0	97.0	98	[146]
	XGB	96.2	96	96	96	
	AdaB	50.5	61	51	51	
	GB	94.53	95	95	95	
	ANN	92.51	92	93	92	
	V. RNN	90.53	90	91	90	
	LSTM	91.23	90	91	90	
	DT	94.23	95	95	95	
	KNN	96.59	97	97	97	
	NB	80.67	84	81	81	
Proposed Dataset	GB	84.1	84.1	84.2	84.1	[147]
	RFs	83.9	83.9	84.1	83.9	
	Bagging	83	83	83.1	83	
	XGB	80.4	80.5	80.4	80.4	
	AdaBoost	77.2	77.3	77.3	77.3	
	DT	76.9	77	77	77	
	MLP	67.6	68.7	67.8	67.8	
	LSVM	65	65.7	65.1	64.9	
	NLSVM	63	63.3	63.2	62.8	
	LR	59.6	60.2	59.8	59.4	
	KNNs	58.9	60.1	59.2	58.9	
	GNB	56.1	59.4	55.4	45.2	
House A	Bernoulli NB	78.7	64	-	-	[148]
	Decision Tree	88	79.4	-	-	
	Logistic Regression	81.4	69.2	-	-	
	KNN	75.8	64.9	-		
House B	Bernoulli NB	95.9	79.4	-		
	Decision Tree	97.2	86.4	-		
	Logistic Regression	96.5	82.7	-		
	KNN	93.1	79.8	-		
UCI HAR	SVM-AdaBoost	99.9			99.9	[149]
	k-NN-AdaBoost	99.43			99.4	
	ANN-AdaBoost	99.33			99.33	
	NB-AdaBoost	97.24			97.2	
	RF-AdaBoost	99.98			100	
	CART-AdaBoost	99.97			100	
	C4.5-AdaBoost	99.95			100	
	REPTree-AdaBoost	99.95			100	
	LADTree-AdaBoost	98.84			98.8	
HAR Dataset	KNN	90.3				[150]
	CART	84.9				
	BAYES	77				
	RF	92.7				
HAPT Dataset	KNN	89.2				
	CART	80.2				
	BAYES	74.7				
	RF	91				
	ET	91.7				
	Proposed Method	92.6				

### 6.4. Deep Learning Applied to Human Activity Recognition Dataset

Implementations based on Deep Learning have become very useful for the identification of activities of daily life, especially those that include image processing [151,152] (see Table 6). Some relevant results of the literature review are detailed below. Wan [153], makes use of the UCI-HAR and PAMAP2 Dataset, implementing algorithms such as CNN, LSTM, BLSTM, MLP, SVM, in which the good results of CNN network implementation are shown with 92.71% and 91% respectively. Akula [154], configures its Dataset to which it applies the algorithms LBP-Naive Bayes, HOG-Naive Bayes, LBP-KNN, HOG-KNN, LBP-SVM, HOF-SVM obtaining better results with the implementation of HOF -SVM with 85.92% accuracy.

He [155], implements DeepConvLSTM, CNN in the UCI-HAR, and Wealky Datasets, showing good results of the implementation of Deep learning with 94.77% and 92.31% respectively. Long [156] in turn uses the Opportunity and Uni-MiB-SAHR Dataset with the algorithms HC, CBH, CBS, AE, MLP, CNN, LSTM, Hybrid, ResNet, and ARN were the results of RNA of 90.29% and 76.39%. Bozkurt [157], for his part, only analyzes the UCI-HAR Dataset, KNN, SVM, HMM + SVM, SVM + KNN, Naive Bayes, Logistic Regression, Decision Tree, Random Forest, MLP, DNN, LSTM, CNN + LSTM, CNN + BiLSTM, Inception + ResNet, the result of the DNN algorithm is shown with an accuracy of 96.81%.

Mekruksavanich [158], uses the Utwente Dataset and PAMAP2, applying the Naive Bayes, SVM, Deep Stacked Autoencoder, CNN-BiGRu techniques, showing better results with this last technique described. Papagiannaki [159] used the FrailSafe dataset with the implementation of CNN networks with an accuracy of 91.84%. Liciotti [139] uses techniques such as LSTM, Bi-LSTM, Casc-LSTM, ENs2-LSTM in the CASAS group dataset to show the dynamics of processes based on deep learning. Hassan [160], applied ANN, SVM and DBN in a proposal dataset for the development of a robust human activity recognition system based on the smartphone sensors’ data, obtaining the following accuracy results ANN 89.06%, SVM 94.12% and DBN 95.85%.

**Table 6:** Deep Learning Techniques results.

Dataset	Technique	Metrics				References
		Accuracy	Precision	Recall	F-Measure	
Uci Har	CNN	92.71	93.21	92.82	92.93	[154]
	LSTM	89.01	89.14	88.99	88.99	
	BLSTM	89.4	89.41	89.36	89.35	
	MLP	86.83	86.83	86.58	86.61	
	SVM	89.85	90.5	89.86	89.85	
PAMAP2	CNN	91.00	91.66	90.86	91.16	
	LSTM	85.86	86.51	84.67	85.34	
	BLSTM	89.52	90.19	89.02	89.4	
	MLP	82.07	83.35	82.17	82.46	
	SVM	84.07	84.71	84.23	83.76	
Propio Infrared Images	LBP-Naive Bayes	42.1	-	-	-	[155]
	HOG-Naive Bayes	77.01	-	-	-	
	LBP-KNN	53.261	-	-	-	
	HOG-KNN	83.541	-	-	-	
	LBP-SVM	62.34	-	-	-	
	HOF-SVM	85.92	-	-	-	
Uci Har	DeepConvLSTM	94.77	-	-	-	[156]
	CNN	92.76	-	-	-	
Weakly Dataset	DeepConvLSTM	92.31	-	-	-	
	CNN	85.17	-	-	-	
Opportunity	HC	85.69	-	-	-	[157]
	CBH	84.66	-	-	-	
	CBS	85.39	-	-	-	
	AE	83.39	-	-	-	
	MLP	86.65	-	-	-	
	CNN	87.62	-	-	-	
	LSTM	86.21	-	-	-	
	Hybrid	87.67	-	-	-	
	ResNet	87.67	-	-	-	
	ARN	90.29	-	-	-	
UniMiB-SAHR	HC	21.96	-	-	-	
	CBH	64.36	-	-	-	
	CBS	67.36	-	-	-	
	AE	68.39	-	-	-	
	MLP	74.82	-	-	-	
	CNN	73.36	-	-	-	
	LSTM	68.81	-	-	-	
	Hybrid	72.26	-	-	-	
	ResNet	75.26	-	-	-	
	ARN	76.39	-	-	-	
Uci Har	KNN	90.74	91.15	90.28	90.48	[158]
	SVM	96.27	96.43	96.14	96.23	
	HMM+SVM	96.57	96.74	06.49	96.56	
	SVM+KNN	96.71	96.75	96.69	96.71	
	Naive Bayes	77.03	79.25	76.91	76.72	
	Logistic Regression	95.93	96.13	95.84	95.92	
	Decision Tree	87.34	87.39	86.95	86.99	
	Random Forest	92.30	92.4	92.03	92.14	
	MLP	95.25	95.49	95.13	95.25	
	DNN	96.81	96.95	96.77	96.83	
	LSTM	91.08	91.38	91.24	91.13	
	CNN+LSTM	93.08	93.17	93.10	93.07	
	CNN+BiLSTM	95.42	95.58	95.26	95.36	
	Inception+ResNet	95.76	96.06	95.63	95.75	
Utwente Dataset	Naive Bayes	-	-	-	94.7	[159]
	SVM	-	-	-	91.6	
	Deep Stacked Autoencoder	-	-	-	97.6	
	CNN-BiGRu	-	-	-	97.8	
PAMAP2	DeepCOnvTCN	-	-	-	81.8	
	InceptionTime	-	-	-	81.1	
	CNN-BiGRu	-	-	-	85.5	
FrailSafe dataset	CNN	91.84	-	-	-	[160]
CASAS Milan	LSTM	76.65	-	-	-	[135]
	Bi-LSTM	77.44	-	-	-	
	Casc-LSTM	61.01	-	-	-	
	ENs2-LSTM	93.42	-	-	-	
CASAS Cairo	LSTM	82.79	-	-	-	
	Bi-LSTM	82.41	-	-	-	
	Casc-LSTM	68.07	-	-	-	
	ENs2-LSTM	83.75	-	-	-	
CASAS Kyoto 2	LSTM	63.98	-	-	-	
	Bi-LSTM	65.79	-	-	-	
	Casc-LSTM	66.20	-	-	-	
	ENs2-LSTM	69.76	-	-	-	
CASAS Kyoto 3	LSTM	77.5	-	-	-	
	Bi-LSTM	81.67	-	-	-	
	Casc-LSTM	87.33	-	-	-	
	ENs2-LSTM	88.71	-	-	-	
Proposal	ANN	89.06	-	-	-	[160]
	SVM	94.12	-	-	-	
	DBN	95.85	-	-	-	

### 6.5. Reinforcement Learning Applied to Human Activity Recognition Dataset

Currently, there is a new trend in reinforcement-based learning processes where it is possible to have systems capable of learning by themselves from punishment and reward schemes, defined by behavioral psychology. It has been introduced in this new line of work for HAR. Which this review shows three highly relevant works (see Table 7). Ber-lin [161], made implementations in the Weizmann and KTH Datasets through the implementation of Spiking Neural Network showing promising results 94.44% and 92.50%. Lu [162] uses the DoMSEV Dataset using the Deep-shallow algorithm with an accuracy of 72.9% and Hossain [163], Pop used a new Dataset to which they implemented the Deep Q-Network algorithm with an accuracy of 83.26%.

**Table 7:** Reinforcement Learning Techniques results.

Dataset	Technique	Metrics	References
		Accuracy	
Weizmann datasets	Spiking Neural Network	94.44	[161]
KTH datasets		92.50	
DoMSEV	Deep-Shallow	72.9	[162]
Proposal	Deep Q-Network (DQN)	83.26	[163]
S.Yousefi-2017	Reinforcement Learning Agent Recurrent Neural Network with Long Short-Term Memory	80	[156]
FallDeFi		83	
UCI HAR	Reinforcement Learning + DeepConvLSTM	98.36	[164]
Proposal		79	[165]
UCF-Sports	Q-learning	95	[166]
UCF-101		85	
sub-JHMDB		80	
MHEALTH	Cluster-Q learning	94.5	[167]
PAMAP2		83.42	
UCI HAR		81.32	
MARS		85.92	
DataEgo	LRCN	88	[168]
Proposal	Mask Algorithm	96.02	[169]
Proposal	LSTM-Reinforcement Learning	90.50	[169]
Proposal	Convolutional Autoencoder	87.7	[170]

### 6.6. Metaheuristic Algorithms Applied to Human Activity Recognition Dataset

In the review of the state of the art, it was possible to identify different metaheuristic techniques that contribute to the identification of different algorithms. Among the most evident results are applications of Genetic Algorithms with the following results 96.43% [171], 87.5 [172], 95,71 [173], 99.75 [174], 98.00 [175] and 98.96 [175]. In many solutions, hybrid systems or new algorithms proposed by the authors are used, see Table 8.

**Table 8:** Metaheuristic Learning Techniques results.

Dataset	Technique	Metrics	References
		Accuracy	
Cifar-100	L4-Banched-ActionNet + EntACS + Cub-CVM	98.00	[175]
Sbharpt	Ant-Colony, NB	98.96	[176]
Ucihar	Bee swarm optimization with a deep Q-network	98.41	[177]
Motionsense	Binary Grey Wolf Optimization	93.95	[178]
Mhealth		96.83	
Uci Har	Genetic Algorithms-SVM	96.43	[171]
Ucf50	Genetic Algorithms-CNN	87.5	[172]
Sbhar	GA-PCA	95,71	[173]
Mnist	GA-CNN	99.75	[174]
Cifar-100	Genetic Algorithms-SVM	98.00	[175]
Sbharpt	Genetic Algorithms-CNN	98.96	[176]

### 6.7. Transfer Algorithms Applied to Human Activity Recognition Dataset

Transfer Learning TL transfers the parameters of the learned and trained model to a new model to help the training of the new model. Considering that most of the data or tasks are related, through transfer learning, the learned model parameters can be shared with the new model in a certain way to speed up and optimize the model learning efficiency. The basic motivation of TL is to try to apply the knowledge gained from one problem to a different but related problem, see Table 9.

**Table 9:** Transfer Learning Techniques results.

Dataset	Technique	Metrics				References
		Accuracy	Precision	Recall	F-Measure	
CSI	KNN	98.3	-	-	-	[179]
	SVM	98.3	-	-	-	
	CNN	99.2	-	-	-	
Opportunity	KNN+PCA	60	-	-	-	[180]
	GFK	59	-	-	-	
	STL	65	-	-	-	
	SA-GAN	73	-	-	-	
USC-HAD	MMD	80	-	-	-	[181]
	DANN	77	-	-	-	
	WD	72	-	-	-	
Proposal	KNN-OS	79.84	85.84	91.88	88.61	[182]
	KNN-SS	89.64	94.41	94.76	94.52	
	SVM-OS	77.14	97.04	79.23	87.09	
	SVM-SS	87.5	94.39	92.61	93.27	
	DT-OS	87.5	94.61	92.16	93.14	
	DT-SS	91.79	95.19	96.26	95.71	
	JDA	86.79	92.71	93.07	92.89	
	BDA	91.43	95.9	95.18	95.51	
	IPL-JPDA	93.21	97.04	95.97	96.48	
	KNN-OS	79.84	85.84	91.88	88.61	
Wiezmann Dataset	VGG-16 MODEL	96.95	97.00	97.00	97.00	[183]
	VGG-19 MODEL	96.54	97.00	97.00	96.00	
	Inception-v3 Model	95.63	96.00	96.00	96.00	
PAMAP2	DeepConvLSTM	-	-	-	93.2	[184]
Skoda Mini Checkpoint		-	-	-	93	
Opportunity	PCA	66.78	-	-	-	[185]
	TCA	68.43	-	-	-	
	GFK	70.87	-	-	-	
	TKL	70.21	-	-	-	
	STL	73.22	-	-	-	
	TNNAR	78.4	-	-	-	
PAMAP2	PCA	42.87	-	-	-	
	TCA	47.21	-	-	-	
	GFK	48.09	-	-	-	
	TKL	43.32	-	-	-	
	STL	51.22	-	-	-	
	TNNAR	55.48	-	-	-	
UCI DSADS	PCA	71.24	-	-	-	
	TCA	73.47	-	-	-	
	GFK	81.23	-	-	-	
	TKL	74.26	-	-	-	
	STL	83.76	-	-	-	
	TNNAR	87.41	-	-	-	
UCI HAR	CNN-LSTM	90.8	-	-	-	[186]
	DT	76.73	.	-	-	[187]
	RF	71.96	-	-	-	
	TB	75.65	-	-	-	
	TransAct	86.49	-	-	-	
Mhealth	DT	48.02	-	-	-	
	RF	62.25	-	-	-	
	TB	66.48	-	-	-	
	TransAct	77.43	-	-	-	
Daily Sport	DT	66.67	.	.	.	
	RF	70.38	.	.	.	
	TB	72.86	.	-	-	
	TransAct	80.83	-	-	-	
Proposal	Without SVD (Singular Value Decomposition)	63.13%	-	-	-	[188]
	With SVD (Singular Value Decomposition)	43.13%	-	-	-	
	Transfer Accuracy	97.5%	-	-	-	
PAMAP2	CNN	84.89	-	-	-	[189]
UCI HAR		83.16	-	-	-	
UCI HAR	kNN	77.28	-	-	-	[190]
	DT	72.16	-	-	-	
	DA	77.46	-	-	-	
	NB	69.93	-	-	-	
	Transfer Accuracy	83.7	-	-	-	
UCF Sports Action dataset	VGGNet-19	97.13	-	-	-	[191]
AMASS	DeepConvLSTM	87.46	-	-	-	[192]
DIP		89.08	-	-	-	
DAR Dataset	Base CNN	85.38	-	-	-	[193]
	AugToAc	91.38	-	-	-	
	HDCNN	86.85	-	-	-	
	DDC	86.67	-	-	-	
UCI HAR	CNN_LSTM	92.13	-	-	-	[194]
	CNN_LSTM_SENSE	91.55	-	-	-	
	LSTM	91.28	-	-	-	
	LSTM_DENSE	91.40	-	-	-	
ISPL	CNN_LSTM	99.06	-	-	-	
	CNN_LSTM_SENSE	98.43	-	-	-	
	LSTM	96.23	-	-	-	
	LSTM_DENSE	98.11	-	-	-	

## 7. Conclusions

The objective of this systematic literature review article is to provide HAR researchers with a set of recommendations, among which the different data sets that can be used depending on the type of research are highlighted. For the development of this analysis, different data sources were considered in an observation window between the years 2017 and 2021. Among the most representative databases, IEEE Xplorer can be highlighted with 256 articles, far surpassing other specialized databases such as Scopus, Science Direct, Web of Science, and ACM.

It is important to specify that 47% of the publications are due to proceedings of congresses or conferences and 36% to the specialized journal. Discriminating the quartiles where the articles are published, it is important to highlight that although the vast majority of publications are indeed focused on conference proceedings that do not have a specific category, 36% of the publications that were made in journals were are mostly in the first two quartiles Q1 and Q2.

In this article, technical analysis of different types of datasets that are used for experimentation processes with HAR was carried out. It should be noted that the creation of new data sets has increased. Some traditional approaches related to the use of indoor datasets based on the WSU Casas project remain. Also, public repositories such as UCI Machine learning have provided sets widely used in the literature such as Opportunity and UCI HAR. It should be noted that the processing of images and videos to the dataset has also been increased, allowing the application of different cutting-edge techniques, such as Weakly Dataset and UniMiB-SAHR.

In this review, different data processing approaches that have been used in this area of knowledge were used. For the specific case of supervised learning, the usability of algorithms based on decision trees such as RandomForest, Naive Bayes, and Support Vector Machine stands out. Regarding unsupervised learning, in most of the analyzed works, the use of techniques such as Spectral Clustering, Single Linkage, Ward Linkage, Average Linkage and K-medioids. Using ensembled learning, it was possible to demonstrate the use of different sets of techniques that allowed improving the results of the experiments, among which those based on classification and grouping can be highlighted. Another modern and widely used approach is the use of DeepLearning focused on datasets with massive image processing requirements, where the use of the following LSTM algorithms stands out, Bi-LSTM, Casc-LSTM, ENs2-LSTM. Other approaches based on Reinforcement learning use resources such as Q-learning and Cluster-Q with learning, in the experimentation processes. The metaheuristic-based approach shows the usability of different algorithms, among which the following stand out: L4-Banched-ActionNet+EntACS+Cub-CVM, Ant-Colony, N.B Bee swarm optimization with a deep Q-network and Genetic Algorithms.

It is important to point out that due to the high demand for data and information processing, it becomes increasingly necessary to implement techniques capable of improving performance and results, such as those based on Reinforcement Learning and Transfer Learning. Another challenge found in the literature is the processing of multi-occupancy datasets that make the use of computational resources and the identification of activities more expensive.

## 8. Future Works

Among the future works that can be implemented after this systematic review of the literature, the real-time analysis of the dataset not only with data from sensors but also images and sound, among which algorithms based on Reinforcement Learning and Transfer Learning can be highlighted. provide a wide range of competitive solutions, adding multi-occupancy in data sets.
